# Return of warm conditions in the southeastern Bering Sea: Physics to fluorescence

**DOI:** 10.1371/journal.pone.0185464

**Published:** 2017-09-28

**Authors:** P. J. Stabeno, J. T. Duffy-Anderson, L. B. Eisner, E. V. Farley, R. A. Heintz, C. W. Mordy

**Affiliations:** 1 NOAA Pacific Marine Environmental Laboratory, Seattle, Washington, United States of America; 2 NOAA Alaska Fisheries Science Center, Seattle, Washington, United States of America; 3 NOAA Alaska Fisheries Science Center, Juneau, Alaska, United States of America; 4 Joint Institute for the Study of the Atmosphere and Ocean, University of Washington, Seattle, Washington, United States of America; Helmholtz-Zentrum fur Ozeanforschung Kiel, GERMANY

## Abstract

From 2007 to 2013, the southeastern Bering Sea was dominated by extensive sea ice and below-average ocean temperatures. In 2014 there was a shift to reduced sea ice on the southern shelf and above-average ocean temperatures. These conditions continued in 2015 and 2016. During these three years, the spring bloom at mooring site M4 (57.9°N, 168.9°W) occurred primarily in May, which is typical of years without sea ice. At mooring site M2 (56.9°N, 164.1°W) the spring bloom occurred earlier especially in 2016. Higher chlorophyll fluorescence was observed at M4 than at M2. In addition, these three warm years continued the pattern near St. Matthew Island of high concentrations (>1 μM) of nitrite occurring during summer in warm years. Historically, the dominant parameters controlling sea-ice extent are winds and air temperature, with the persistence of frigid, northerly winds in winter and spring resulting in extensive ice. After mid-March 2014 and 2016 there were no cold northerly or northeasterly winds. Cold northerly winds persisted into mid-April in 2015, but did not result in extensive sea ice south of 58°N. The apparent mechanism that helped limit ice on the southeastern shelf was the strong advection of warm water from the Gulf of Alaska through Unimak Pass. This pattern has been uncommon, occurring in only one other year (2003) in a 37-year record of estimated transport through Unimak Pass. During years with no sea ice on the southern shelf (e.g. 2001–2005, 2014–2016), the depth-averaged temperature there was correlated to the previous summers ocean temperature.

## Introduction

**Fig 1:**
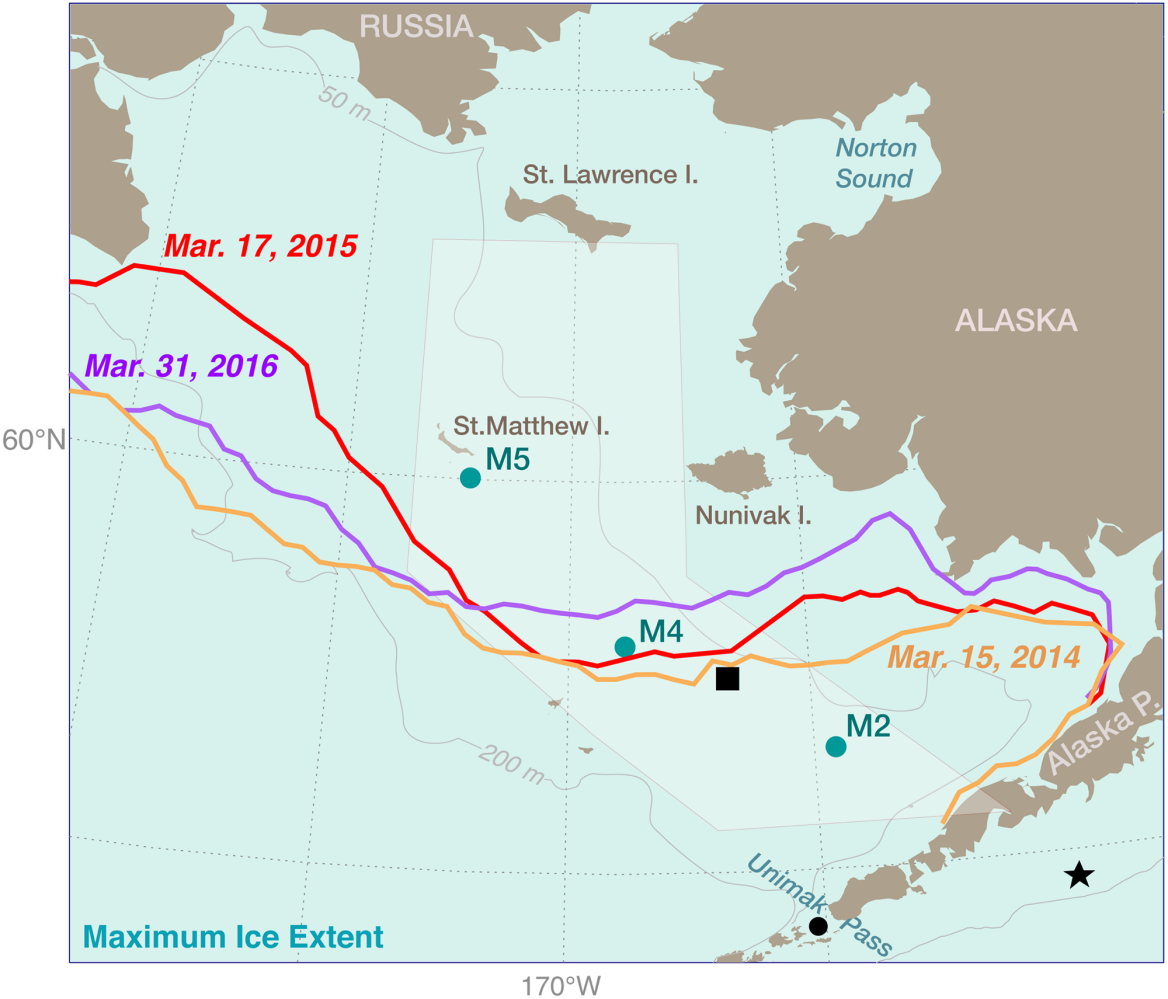
The eastern Bering Sea shelf. Colored lines indicate maximum ice extent in 2014 (orange), 2015 (red), and 2016 (purple). The locations of three long-term moorings (M2, M4, and M5) are shown. The star in the Gulf of Alaska and the square in the Bering Sea are locations of interpolated winds and sea surface temperature used in this manuscript. The pale shading indicates the area where the time series of percent ice cover as a function of date and latitude was calculated. The 50-m, 100-m, and 200-m isobaths are shown.

The Bering Sea is a productive high-latitude sea situated between the Arctic Ocean and the North Pacific (Fig 1). This marine ecosystem produces ~40% of the United States’ catch of fish and shellfish, supports 25 species of marine mammals and 35 million seabirds, and provides three quarters of the subsistence harvest supporting 55,000 Alaska Natives [1]. While the eastern Bering Sea supports a rich and robust ecosystem, it also responds rapidly to climate change (e.g., [2,3]), and, as a subarctic sea, it is predicted to be sensitive to such changes [4].

The eastern Bering Sea is ice-free during the summer and much of the fall. Sea ice usually begins to form on the northern shelf in December, with strong, frigid northerly winds both opening polynyas where sea ice forms and advecting the ice southward [5]. The leading edge melts, cooling and freshening the water column. Typically, ice appears on the southern shelf in January, reaches a maximum in February or March, and is gone by mid-May [6]. In cold years, sea ice advances more than 1000 km from the Bering Strait (66°N) to the Alaskan Peninsula, while in warmer years, ice remains north of 58°N.

Historically, the southeastern Bering Sea has been characterized by high year-to-year variability in sea-ice extent in March and April [7]. This high variability was interrupted in 2001 by a five-year period of low (almost nonexistent on the southern shelf) sea-ice extent and warm ocean temperatures (2001–2005). To improve understanding of how long-term warming can impact this ecosystem, a major study (Bering Sea Project, http://www.nprb.org/bering-sea-project) was designed to explore this ecosystem from 2007 to 2010. Because of the historical record of high inter-annual variability, it was expected that at least one of the project years would also support warmer (less sea ice) conditions. Unfortunately for the Bering Sea Project, the warm period of 2001–2005 was followed by a period of extensive sea ice during spring and cold ocean temperatures over the entire eastern shelf (2007–2012) [7,8]. During the intermediate year, 2006, some ice was observed on the southern shelf and ocean temperatures were near normal. To examine the impact of warming on the ecosystem, data from the Bering Sea Project were combined with observations collected in the early 1990s through 2016 as part of several NOAA programs (e.g., Ecosystem Fisheries Oceanography Coordinated Investigations (EcoFOCI), Bering-Aleutian Salmon International Survey (BASIS), Coastal Ocean Program) and NSF-supported studies (Inner Fronts, Pribilof Domain).

A major finding from the Bering Sea Project is that a transition exists at ~60°N that divides the eastern Bering Sea shelf into northern and southern regions [6]. The southern shelf ecosystem, which supports most of the commercial fisheries, is sensitive to periods of two or more years of no sea ice. Specifically, reduced sea ice in March and April results in warmer (>2°C) than average ocean temperatures. When consecutive ice-free years occurred in 2001–2005 on the southern shelf, there was a profound effect on the ecosystem, including: decrease in the populations of large, lipid-rich zooplankton [7,9,10]; increase in the numbers of small zooplankton [9]; reduced lipid content in young-of-the-year walleye pollock (*Gadus chalcogrammus*) in late summer [7,11]; reduced winter survival of young-of-the-year pollock [12]; and finally, failure of pollock to recruit to the fishery three to four years later. Poor recruitment during the previous warm period (2001–2005) precipitated action by the North Pacific Fisheries Management Council to reduce the Total Allowable Catch of pollock.

At the end of the Bering Sea Project in 2010, it was not known how long the cold period would continue, nor were there well-formed hypotheses of whether the southern Bering Sea would shift back to high year-to-year variability or into another multi-year period (stanza) of warmth. This paper examines the southeastern Bering Sea starting when conditions turned from cold to warm in 2014. Fortunately, EcoFOCI’s and BASIS’ long-term observational plan supported extensive field seasons in the Bering Sea in 2014 (five oceanographic cruises with 79 days at sea) and 2016 (6 oceanographic cruises with 78 days at sea). In 2015, the long-term plan was structured so that most of the EcoFOCI/BASIS research would be in the Gulf of Alaska. Predictions that 2015 would also be warm resulted in additional funding to plan and execute an extensive field season (four cruises with 59 days at sea) in the Bering Sea.

This paper explores the temporal variability on the southern Bering Sea shelf during three consecutive warm years (2014–2016) and compares these years to previous years (1995–2013). Patterns of variability in ocean temperatures and chlorophyll fluorescence are examined at two long-term mooring sites, and related to the presence of ice in March and April. While atmospheric forcing is the primary driver of sea ice on the Bering Sea shelf, evidence is presented that the extremely warm conditions in the Gulf of Alaska in 2015 played a role in limiting the southern extent of ice that year. How chlorophyll fluorescence varies during these years is discussed, as is the surprisingly high and unforeseen accumulation of nitrite in oxygenated waters near St. Matthew Island. These observations reveal similarities and differences in the lower trophic system between the two warm periods: 2001–2005 and 2014–2016.

## Data and methods

Three sources of sea-ice data were used. The first source was the National Ice Center (http://www.natice.noaa.gov/), with data available from 1972 to 2002. The second source (2002–2011) was the Advanced Microwave Scanning Radiometer EOS (https://nsidc.org/data/AE_SI12/versions/3). After October 2011, Special Sensor Microwave/Imager (SSM/I) and Special Sensor Microwave Imager Sounder (SSMIS) from the National Snow and Ice Data Center (ftp://sidads.colorado.edu/pub/DATASETS/nsidc0079_gsfc_bootstrap_seaice/final-gsfc/north/daily) were used. Details are provided by *Stabeno et al*. [7].

Reanalysis data were obtained from the North American Regional Reanalysis (NARR). NARR uses the high-resolution National Centers for Environmental Prediction (NCEP) Eta model (~32 km grid size) and includes additional assimilated parameters to improve the reanalysis product [13]. Reanalysis estimates of winds are available at 3-hr intervals for NARR. The NARR data were provided by the NOAA/OAR/ESRL PSD, Boulder, Colorado, USA, from their web site at http://www.esrl.noaa.gov/psd/.

Data collected from a series of moorings deployed at two biophysical sites (M2 and M4) included temperature (miniature temperature recorders, SBE-37 and SBE-39), salinity (SBE-37), and fluorescence (WET Labs DLSB ECO Fluorometer). At M2, moorings are typically deployed and recovered in May and September, while at M4 one year-long mooring is typically deployed in September. In addition, current measurements were collected at a site in Unimak Pass [14]. All instruments were prepared and the data were processed following the manufacturer’s specification. Conversion of fluorescence to chlorophyll was performed using the nominal relationships provided by the manufacturer. Those relationships are meant to provide a means of comparing the fluorescence measured using different sensors and provide an estimate of the amount of in situ chlorophyll. It is acknowledged that the relationships provided by the manufacturer cannot represent the range of species and physiological states of the cells found in our samples. *Stabeno et al*. [7] discuss details of M2 and M4 mooring designs and data processing, and the calculation of the depth averaged temperature at M2.

Since 2003, the EcoFOCI and BASIS programs have collected discrete oceanographic samples for nutrient analysis, with some variance in the handling and processing of these samples. During EcoFOCI cruises, samples were filtered through 0.45 μm cellulose acetate filters and either analyzed at sea, or returned to NOAA's Pacific Marine Environmental Laboratory for analysis following protocols of *Gordon et al*. [15]. During BASIS, samples collected between 2003 and 2011 were stored frozen without filtration, and analyzed at the University of Washington Marine Chemistry Laboratory (UWMCL) following protocols of *Knap et al*. [16]. In subsequent years, BASIS sampling and analysis followed EcoFOCI protocols. Comparable methods were used at the two laboratories including calibration of labware, preparation of primary and secondary standards, and corrections for blanks and refractive index. Replicate analysis found good agreement between PMEL and UWMCL nutrient protocols, and between filtered and unfiltered frozen samples [17]. In both laboratories, nitrite analysis was completed using segmented flow analysis where nitrite was diazotized with sulfanilamide and coupled with N-(1-naphthyl)-ethylenediamine to form a red dye, and measured with colorimetric detection.

The Prawler is a platform that utilizes wave energy to move an instrument package up the mooring line using a ratcheting device, and then collects data during a free-fall down the mooring line. The rapidity of cycling is dependent upon the available wave energy and settings on the mooring. In May 2016, a Prawler mooring was deployed at M2 (72-m bottom depth) and profiled the upper ~45 m of the water column approximately every two hours. Among the various sensors deployed on the Prawler was a Wet labs ECO FLNTU fluorometer that measured chlorophyll-*a* fluorescence. This instrument was calibrated and the data processed according to manufacturer's specifications.

## Results and discussion

### Patterns of interannual variability

Sea ice is the dominant characteristic of the eastern Bering Sea shelf [6,7], with March and April ice extent setting up the marine ecosystem for the following spring, summer, and autumn. Moorings have been deployed at M2 near the center of the southeastern Bering Sea shelf (Fig 1) almost continuously since 1995 and provide an extensive data set to examine how March/April sea ice impacts the ocean over the southern shelf.

**Fig 2:**
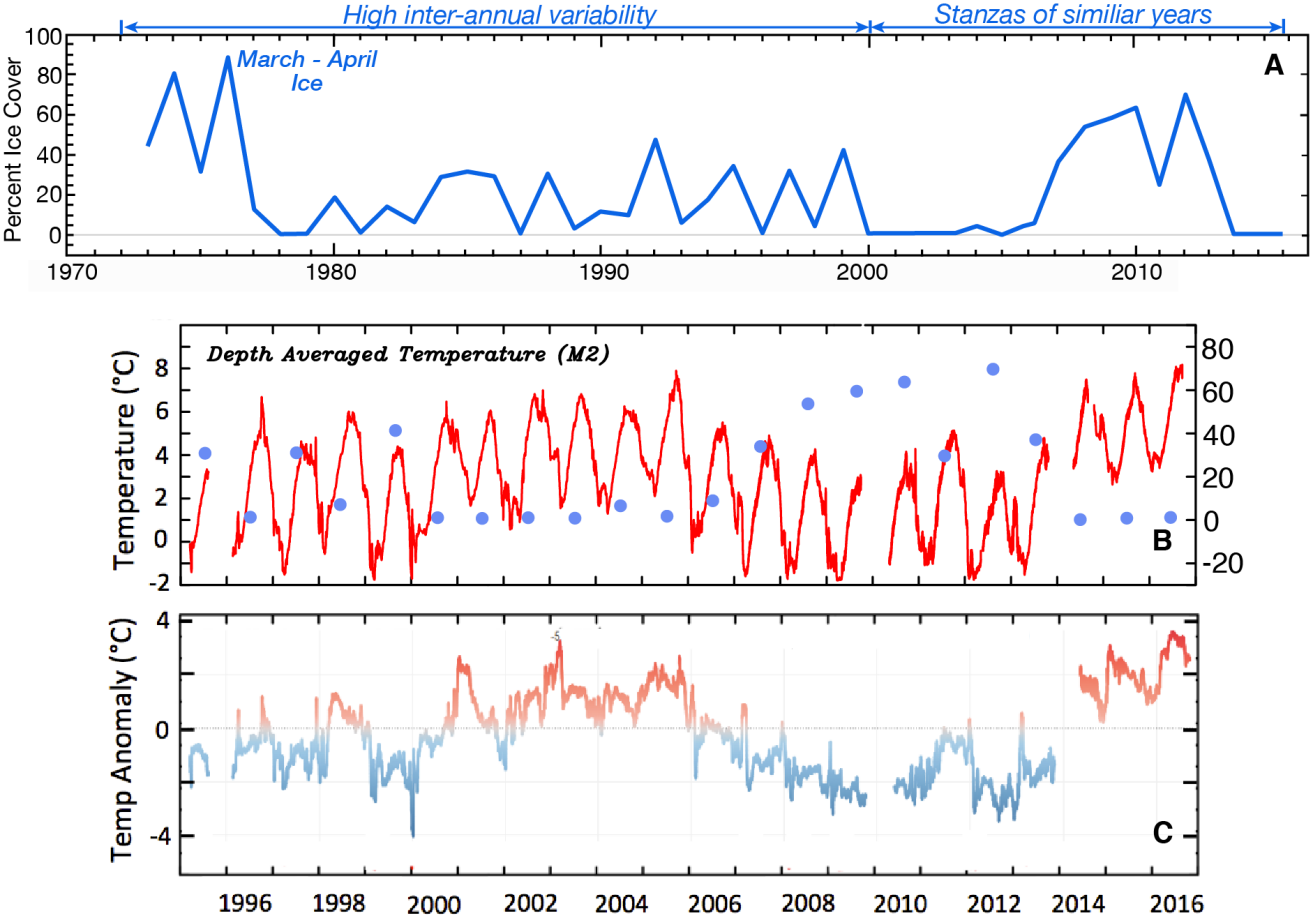
Ice and temperature at M2. (A) The average percent of areal ice cover in a 2° × 1° box (163–165°W, 56.5–57.5°N) around M2 during March–April. (B) The depth-averaged hourly temperature at M2. The circles are a replotting of the percent ice cover data found in (A). (C) The depth-averaged temperature anomaly (relative to 1995–2009) at M2.

The interannual variability in spring ice cover discussed in the introduction is evident in the time series of mean ice concentration in a box roughly 100 km on a side centered at M2 (Fig 2A). The first warm/low-ice period (2001–2005) ended in 2006. Even though the areal average ice cover was only slightly higher in 2006 (~5%) than that in 2004 (~2%), average temperature was ~1.2°C cooler. Even small amounts of ice can efficiently cool the water column through melting (latent heat flux). In addition, sea ice persisted ~50 km north of the M2 box through 11 May 2006 indicative of cold atmospheric conditions. Sea ice was less extensive in 2011 than in the other years of the recent (2007–2013) cold period. During this year, sea ice was primarily present at M2 in March, which resulted in the depth-average ocean temperatures at the mooring being near average rather than colder than average (Fig 2C). Both 2012 and 2013, however, had extensive sea ice in the vicinity of M2 in April, resulting in cold ocean temperatures. The recent cold period ended in 2013 and was followed by three warm years, 2014, 2015, and 2016. Temperatures in 2015 and 2016 were particularly warm (daily depth-averaged temperature anomalies approaching 4°C at times, Fig 2C). Note that ocean temperatures were warm even in winter, with the minimum temperature in January through March of 2015 and 2016 approximately equal to the warmest summer temperatures in 2009 and 2012 (Fig 2B).

**Fig 3:**
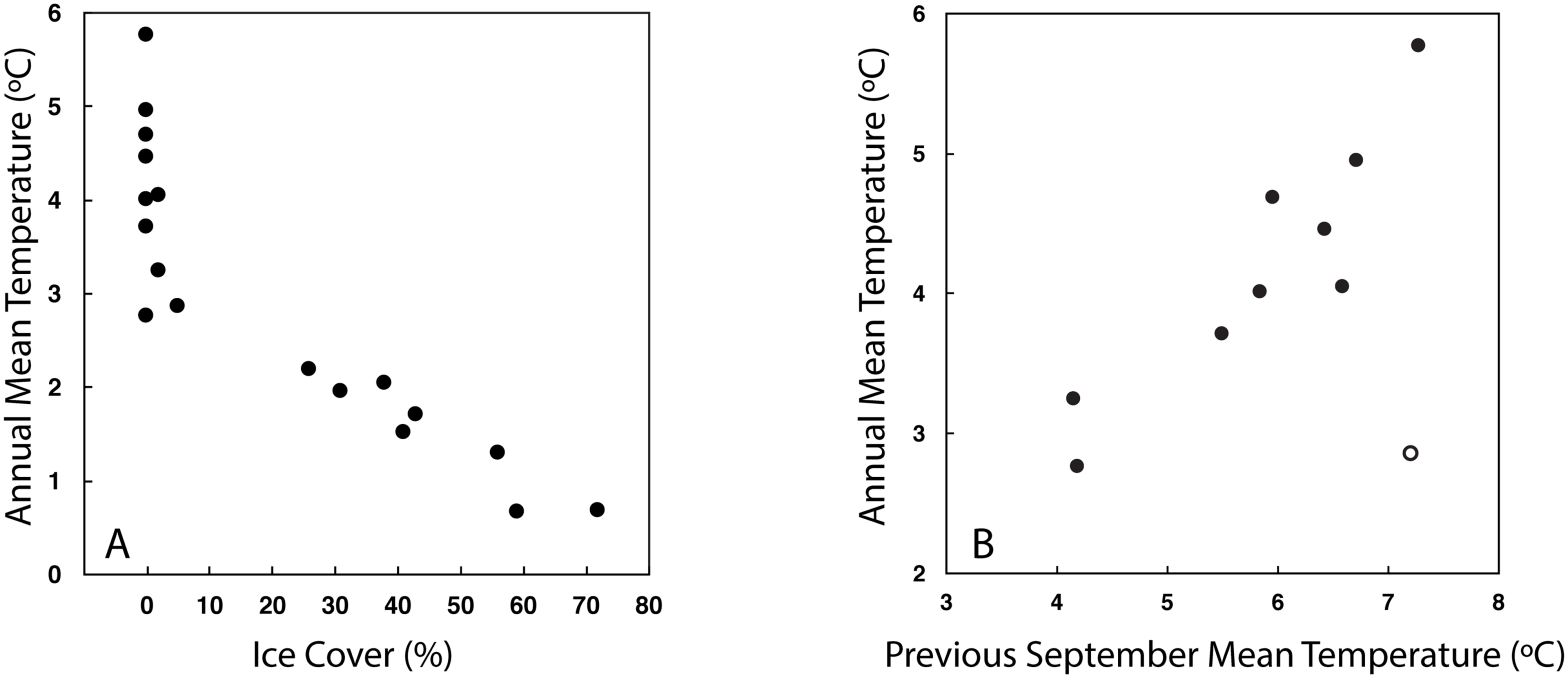
Ocean temperature and sea ice persistence. (A) Mean annual depth-averaged temperature at M2 as a function of average percent of areal ice cover in a 2° × 1° box (163–165°W, 56.5–57.5°N) around M2 during March–April (i.e., Fig 2A). Years with a data gap of more than 100 days (e.g., 1995, 1996, 2010, and 2014) are not included. Autumn data for 2009, 2013, and 2016 were estimated by using the relationship between September average ocean temperature (ST) and October-December (autumn) average temperature (AT): AT = 0.89 ST—0.60, R^2^ = 0.85. (B) Annual average temperature for years with little or no ice in March-April as a function of the monthly mean temperature in September of the previous year. The open circle represents the average annual temperature in 2006 versus September mean temperature in 2005.

Over the southern Bering Sea shelf, the presence/absence of sea ice in March and April largely determines the depth-averaged ocean temperature (Figs 2 and 3A), not only for spring, but for spring through autumn. Years in which there was no sea ice in March and April were strongly associated with warm (>3.5°C) average-annual ocean temperatures (Fig 3A). This year-long response is a result of seasonal factors that can combine together to create a warm or cold year. For instance, above average ocean temperatures in January and February can delay the arrival of ice [18], and the lack of ice in March and April result in above average ocean temperatures in those two months. Finally, the above average ocean temperatures in May persist through October, and it is only in November with the return of winter conditions that heat can be quickly removed from the water column. The inverse pattern occurs when there is extensive ice in March and April: cold ocean conditions in January and February permit arrival of ice, presence of ice in March and April coincide with cold ocean temperatures and these cold ocean temperatures persist for the remainder of the year.

Areal ice concentrations in March and April were inversely related to annual ocean temperatures (Fig 3A). In years when ice was present (>10%), the depth-average ocean temperature at M2 decreased by ~2°C as ice cover increased from 20 to 70%. In ice-free years, the range in annual temperature, however, was even greater, >3°C. Comparing the annual mean temperature during years with little or no ice to the previous year’s mean September temperature (September has the highest depth-averaged temperature) reveals a significant relationship (R^2^ = 0.86, p<0.01) if 2006 is not included in the analysis. If 2006 is included, then the correlation is not significant (p>0.05), supporting the claim that even a low percentage of areal ice cover, especially if it is in April, can significantly cool the water column.

### Temperature in 2014–2016

To investigate the water column in more detail during the most recent warm period, we examined the near-surface and near-bottom temperatures at M2 and M4 in 2014–2016. At M2, the near-surface thermistors were at 4 m in May through September and at 11 m the remainder of the year, while at M4 the near-surface thermistor was at 11 m for the entire year. Near-bottom thermistors were ~10 m off the bottom for all moorings for each year. Both M2 and M4 were deployed in the middle domain near the 70-m isobath (Fig 1). The middle domain (water depth 50–100 m) on the eastern Bering Sea shelf is characterized by a two-layer structure from mid-spring through early autumn, with a surface mixed layer typically 20–25 m deep [19, 20]. At its maximum extent, sea ice barely covered M4 in 2014 and 2015, but in neither year did it reach M2 (Fig 1). Maximum ice extent in 2016 was less than in the previous two years, not reaching M4 let alone M2.

**Fig 4:**
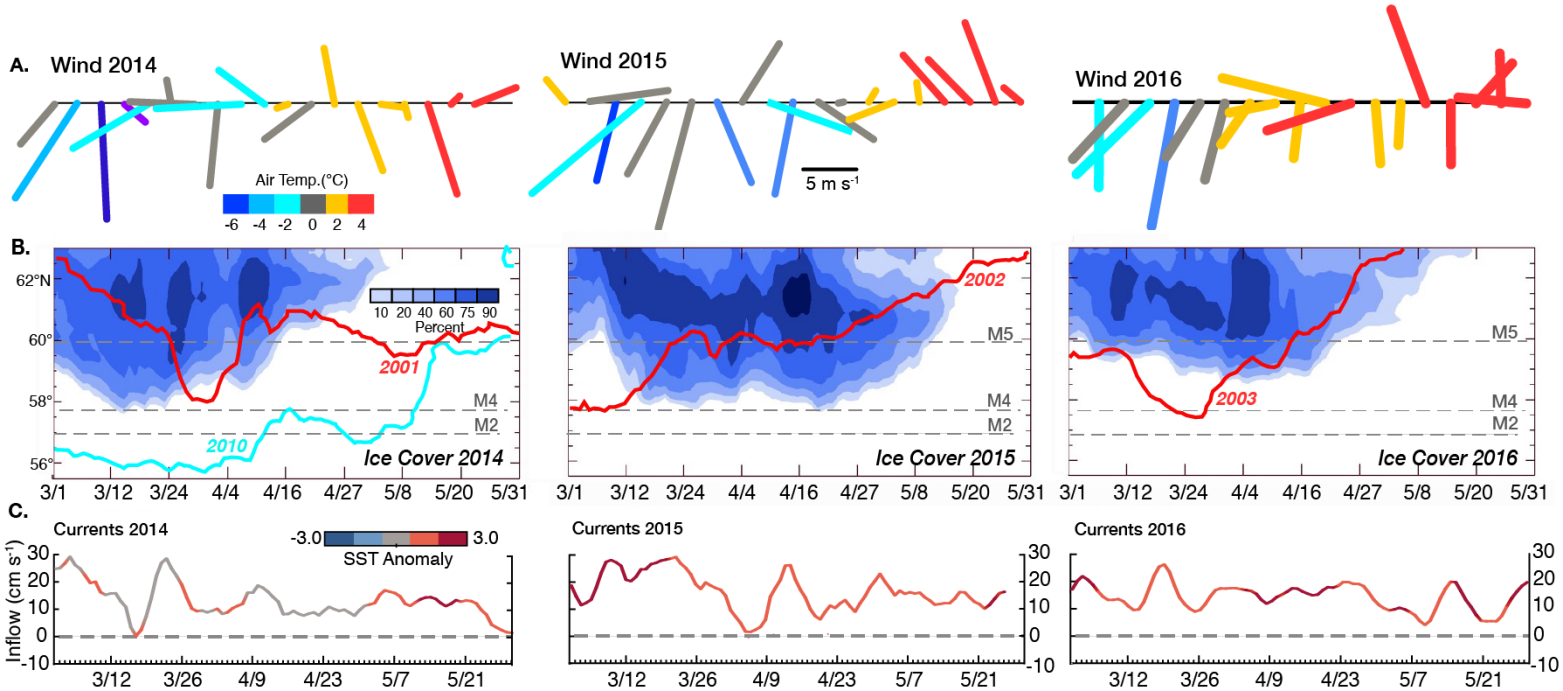
Winds, currents and sea ice. Panels (A)–(C) are March–May time series from 2014 (left), 2015 (middle), and 2016 (right). (A) 5-day average winds at 57.32°N, 166.32°W. Vectors are color-coded according to air temperature. (B) Time series of contours of daily areal ice concentration for 0.25° latitude bands in the light-shaded area in Fig 1. The latitudes of the two long-term moorings on the southern Bering Sea shelf are indicated with dashed lines, as is M5 in the transition zone between the southern and northern shelf. Daily maximum ice extent is indicated for 2010 (blue) and 2001, 2002, and 2003 (red). (C) Daily mean derived currents through Unimak Pass. Lines are color-coded by SST anomaly upstream near the Shumagin Islands (star in Fig 1, 54.625°N, 161.125°W). Positive indicates inflow into the Bering Sea.

Fig 1 shows an areal view of maximum ice extent, while Fig 4A shows the temporal variability of ice concentration and extent over the middle shelf (pale hexagon in Fig 1) during these three warm years. In these three years, sea ice remained largely north of M4 (57.9°N), as did the sea ice in 2001, 2002, and 2003 (red lines Fig 4B). The largest differences between the two periods was that the ice in March 2003 was more extensive than in March 2016 and ice persisted at M4 from mid-March to early June 2015 (i.e., longer than the other years). For comparison to ice extent in a cold year, the time series of the latitude of the ice edge for 2010 is shown (blue line in Fig 4B, first panel).

Ice extent is determined largely by atmospheric forcing [5]. Frigid winds out of the north or east open polynyas (which permit ice formation), transport the ice southward and cool the water column into which ice is being advected. In the first half of March in each year (2014–2016), cold southward winds (Fig 4A) forced the ice southward. After March 21 in 2014 and 2016 there were no cold northerly or northeasterly winds, while in 2015 cold northerly winds persisted into mid-April. These winds in 2015, however, did not result in extensive sea ice over the southern shelf, but did help to prolong the presence of ice near M4 (Fig 4B).

**Fig 5:**
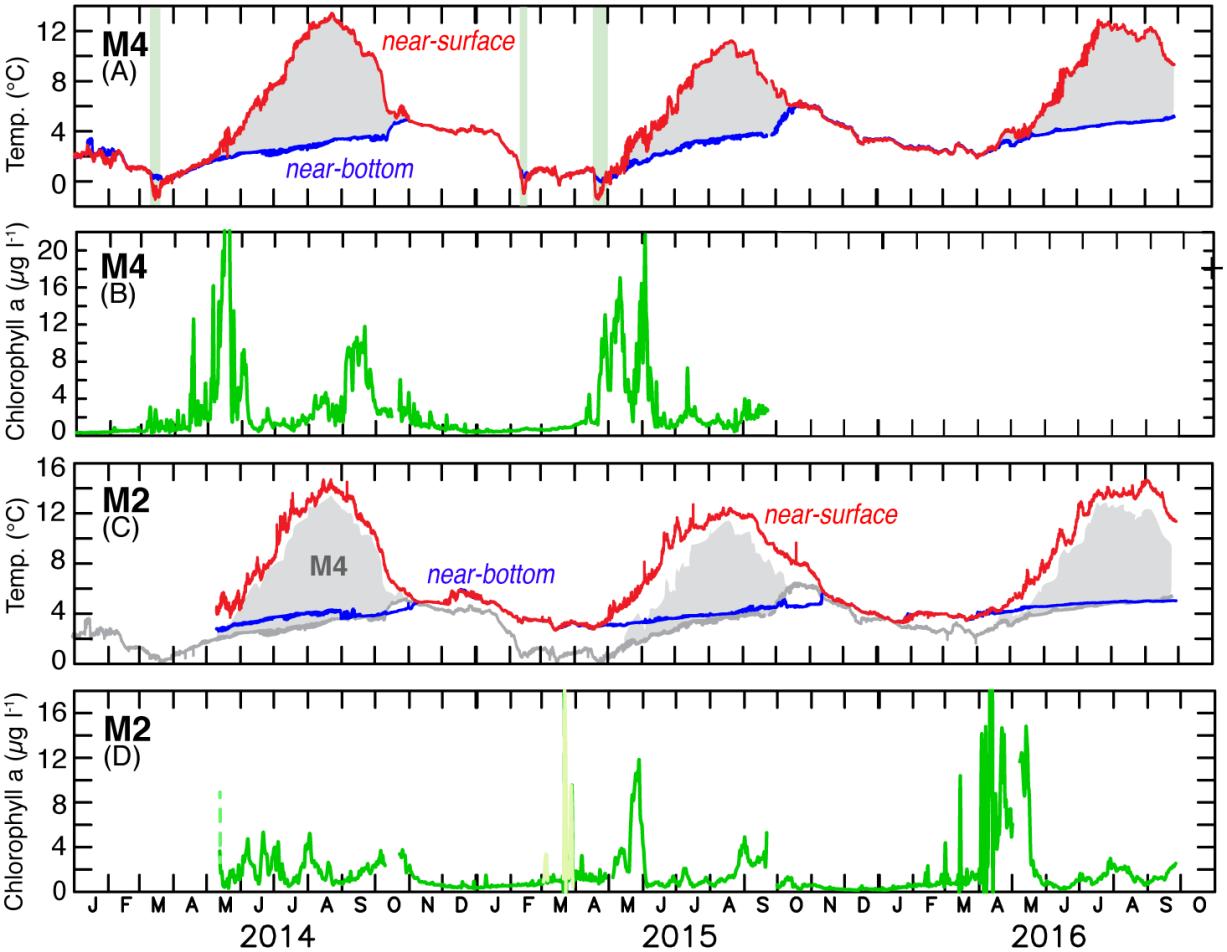
Moored biophysical measurements. The time series in 2014–2016 of near-surface and near-bottom temperature at (A) M4 and (C) M2, and chlorophyll fluorescence at ~11 m at (B) M4 and (D) M2. All data have been low-pass filtered with a 35-hour Lancsoz filter. The pale green stripes in (A) indicate the presence of sea ice (>15% ice cover). Note that (A) and (C) have the same aspect ratio, but the scale is shifted by 2°C, and that (B) and (D) have different scales. The gray in (C) indicates the range of temperature at M4 and is to ease comparison between M2 and M4. Fluorescence, indicated by pale green in (D), was a short period of extremely high variability.

Melting ice rapidly cools only the surface waters, while cooling of near-bottom waters occurs only after vertical mixing of the water column [18]. The three largest (longest) occurrences of ice at M4 (one in March 2014, the others in February and April 2015; Fig 5A) show this cooling of near-surface temperatures caused by melting ice, with the bottom temperatures largely unaffected. Coincident with the surface cooling is freshening of the near-surface water, which stabilizes the water column. Historically, if ice is present at a site for more than a few weeks, winds and tides mix the water, resulting in a cold (~ -1.7°C) and less saline water column [18].

After the first ice event in mid-March 2014, ice remained north of M4 and near-surface temperatures were only slightly cooler at M4 than at M2 through mid-August (Fig 5). In contrast, the third time ice was present at M4 in late April 2015 it persisted for ~10 days resulting in colder (~2°C) surface temperatures at M4 than at M2 throughout the summer. In 2016, even without ice, near-surface temperatures were cooler at M4 than at M2. Bottom temperatures had a similar pattern to near-surface temperatures in the spring, but in all three years, warming of the near-bottom waters was greater at M4 than at M2. This warming was great enough that in the fall, when the water column became well mixed (e.g., when near-surface and near-bottom temperatures were the same), there was no significant difference between M2 and M4 temperatures (Fig 5C).

Near-surface temperatures varied among the three years, with near-surface summer temperatures ~2°C colder in 2015 than in 2014 or 2016. This interannual difference occurred at both mooring sites, even though the depth-averaged temperatures in 2015 at M2 exceeded those in 2014 (Fig 2B). This apparent contradiction between near-surface and depth-averaged temperatures resulted from a deeper (~25%) mixed layer in 2015 than in 2014. In contrast to the surface waters, near-bottom temperatures at M2 were almost identical in the three years. Bottom temperatures at M4, however, were more variable among the years likely as a result of the presence or absence of sea ice.

### Chlorophyll fluorescence and nitrite in 2014–2016

While the most obvious impact of the presence of sea ice after mid-March is cold ocean temperatures, ice also influences the timing of chlorophyll blooms. The spring chlorophyll bloom in years with extensive sea ice in March and April is earlier (April) than years with no ice in March or April [7,8]. In addition, ice associated blooms are often associated with different taxa than open water blooms [8]. How do the blooms in 2014–2016 compare to earlier findings?

In April 2015, the stratification at M4 resulting from melting ice was associated with increased chlorophyll (Fig 5A and 5B). This high chlorophyll may be due to ice-associated phytoplankton taxa comprised of numerous chains of large diatoms; details are described by *Duffy-Anderson et al*. [21]. A similar but weaker event occurred in March 2014. The large values of fluorescence (pale green; Fig 5D) at M2 in late March were highly variable and associated with strong winds. In contrast, the high chlorophyll concentrations at both sites in May and into June in 2014 and at M2 in 2015 were associated with thermal stratification of the water column (Fig 5). At both M2 and M4, periods of higher chlorophyll occurred throughout the summer and were associated with periods of vertical mixing, which introduced nutrients into the surface layer. Chlorophyll fluorescence was substantially higher at M4 than at M2 during the spring bloom period (April/May) in the first two years (the fluorometer at M4 failed in 2016) and during the fall bloom period (September) in 2014 (Fig 5B and 5D). These results are consistent with prior research that indicated that August/September chlorophyll levels in the region near M4 were some of the highest on the shelf in both warm and cold years [22]. Finally, during the autumn, increased chlorophyll occurred with the beginning of fall mixing of the water column.

**Fig 6:**
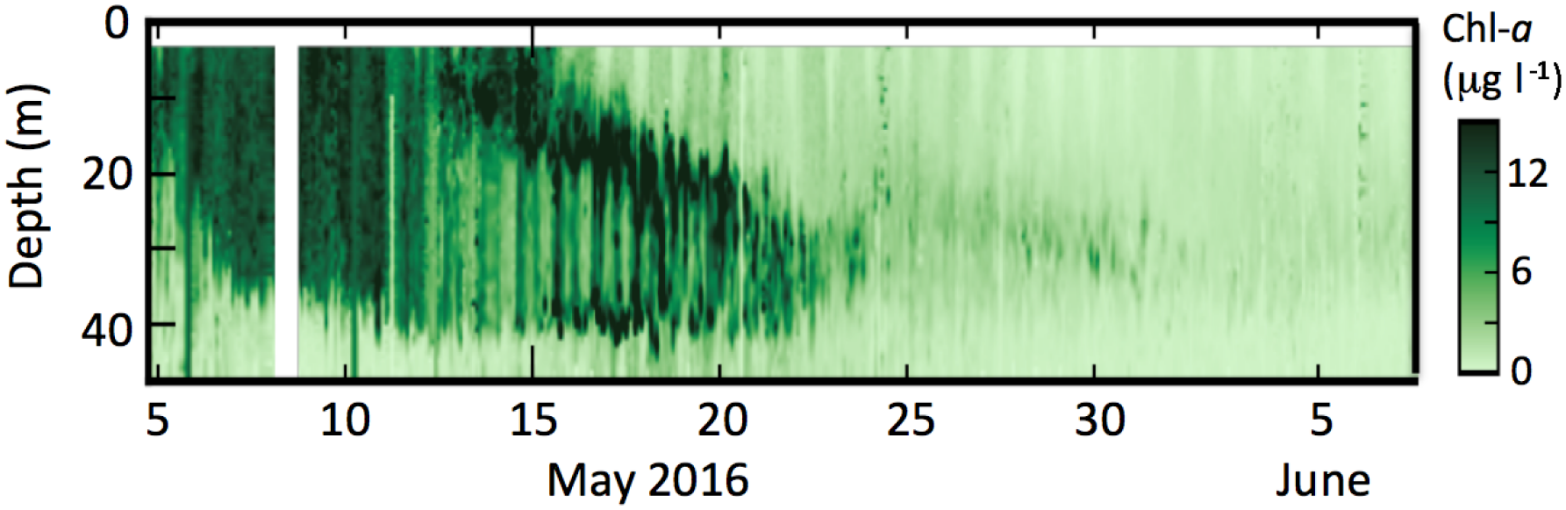
Chlorophyll fluorescence at M2. Data were measured on the Prawler.

In 2016, the fluorometers deployed on both the winter and summer moorings at M2 showed much higher chlorophyll fluorescence values than in the other two years. In addition, the bloom began in mid-April earlier than is typical for a year without sea ice [7,8], likely a result of mid-April stratification of the water column. Unfortunately, the fluorometer at M4 failed, preventing comparison with M2, but this more intense bloom at M2 was captured by a companion profiling-mooring, the Prawler (Fig 6). Upon deployment of the Prawler in the beginning of May, the spring phytoplankton bloom was already underway and concentrations were similar to those measured on the main M2 mooring at 11 m. In mid-May the chlorophyll appeared to sink below 11 m. A band of higher chlorophyll fluorescence concentrations persisted at the interface between the surface and bottom mixed layers. This slightly higher fluorescence along the interface was surprising since subsurface blooms while common in the northern Bering Sea are rarer at M2 [6].

Considerable variability on time scales of less than daily is evident in this time series (Fig 6). In the upper ~15 m of the water column, a diurnal cycle is evident, with minimum florescence during daylight hours (photoinhibition). Deeper in the water column (especially during 15–21 May), a semidiurnal signal is evident. This is likely due to advection by semidiurnal tides [20].

In addition to warmer temperature and later spring blooms during years with reduced sea ice [7,8], other parts of the lower trophic levels also respond differently in warm and cold years. The presence of sea ice is associated with high-energy ice algae, which is source of food for lipid-rich zooplankton [21]. Noting this, it is not surprising that distinct differences between zooplankton populations during the warm period (2001–2005) and the cold period (2007–2013) occurred. Some of the mechanisms that result in other differences between warm and cold stanzas, however, are not clear. One such difference is the presence of high nitrite concentrations during warm years.

**Fig 7:**
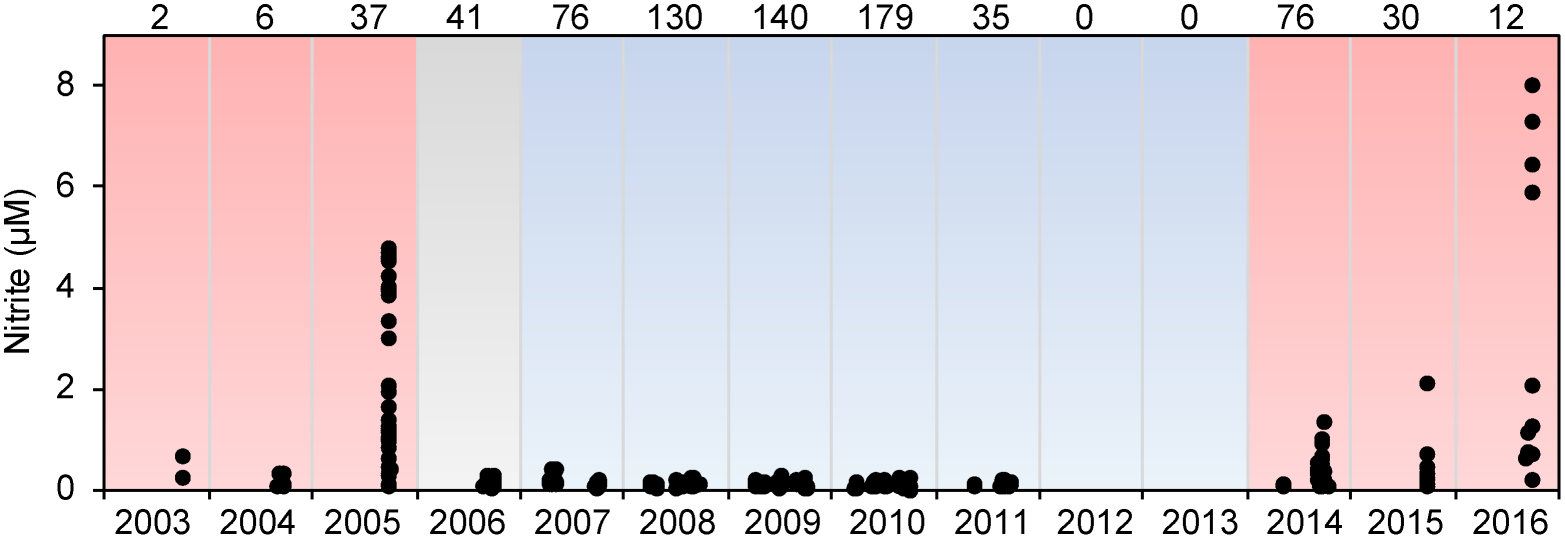
Nitrite. Concentration of nitrite within 12 m of the bottom in the vicinity of St. Matthew Island (59°N– 62°N, 50–100 m water depth). Color shading represents years with predominantly warm (red) or cold (blue) temperature anomalies at the M2 mooring. Numbers along the top indicate the number of stations analyzed.

During warm conditions in 2005, there appeared to have been a disruption in the nitrogen cycle (i.e., nitrification) in the vicinity of St. Matthew Island [17]. Nitrification is the two-step oxidation of ammonium to nitrate. The first, rate-limiting, step is ammonium oxidation to nitrite, followed by nitrite oxidation to nitrate. Thus, nitrite is a short-lived intermediate in the nitrogen cycle and usually does not accumulate in aerobic waters. However, in the summer of 2005, nitrite concentrations southeast of St. Matthew Island exceeded 4 μM (Fig 7). This was a transitory feature that was serendipitously observed by two independent research programs. This feature was not observed during the cold years of 2007–2011, but unusually high (1–8 μM) concentrations were once again observed in 2014–2016 concomitant with warmer conditions. An associated decrease in ammonium concentrations was reported by *Mordy et al*. [17], suggesting that this transitory nitrite pool was due to an interruption of nitrification in the water column. It is unclear why warmer temperatures (or reduced ice cover) on the southern shelf would result in localized uncoupling of the marine nitrogen cycle near St. Matthew Island and what impact such a change would have on the broader ecosystem.

### The role the Gulf of Alaska hot spot played in reducing ice

The strength and persistence of cold northerly winds are the primary factors that determine the southern extent of sea ice, although extremely warm ocean conditions during the previous summer can delay, but not prevent, the advance of sea ice [20]. Despite the duration of cold northerly winds in 2015, ice was not advected south of the M4 mooring. Other factors must have helped limit ice extent. One possible influence was the warm conditions that persisted in the Gulf of Alaska in 2015 [23].

**Fig 8:**
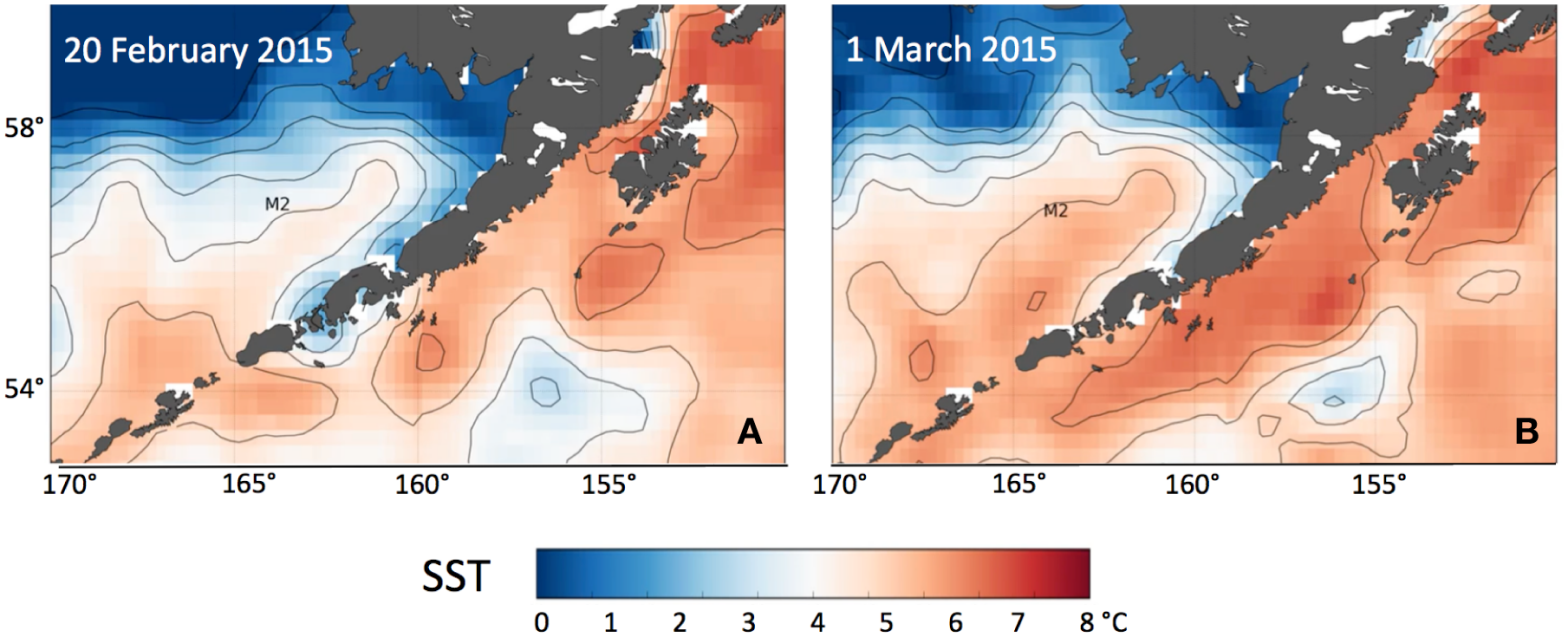
Sea surface temperature. SST for (A) 20 February 2015 and (B) 9 days later on 1 March. NOAA High Resolution SST data provided by the NOAA/OAR/ESRL PSD, Boulder, Colorado, USA, from their web site at http://www.esrl.noaa.gov/psd/. The location of mooring site M2 is indicated.

A pair of sea surface temperature (SST) maps shows the apparent intrusion of warm water into the Bering Sea (Fig 8). In late February cold water is evident north of M2 with ~4°C surface water south of M2. Nine days later, waters north of M2 (e.g., 58°N) remained cold, but south of the mooring SST had warmed by ~1°C. Such warming in mid-winter is only possible through advection.

Water from the Gulf of Alaska enters the Bering Sea through the Aleutian Passes, but the greatest net transport of shelf water is through Unimak Pass [14]. During winter, >0.4 × 10^6^ m^3^ s^−1^ of Gulf of Alaska shelf water flows northward into the Bering Sea through this pass, advecting heat, salt, nutrients and zooplankton onto the southern Bering Sea shelf. In addition, some Gulf of Alaska water that enters the Bering Sea through other Aleutian Passes is advected onto the shelf through Bering Canyon, but the magnitude of this flow appears to be much weaker than that through Unimak Pass [14]. During the winter months, approximately half of the water on the southern Bering Sea shelf is replenished by water from the eastern Bering Sea slope via Unimak Pass and Bering Canyon [14].

Moorings have been deployed in Unimak Pass on multiple occasions (Table 1). Dividing the data set into warm months (May–September) and cold months (October–April) and then calculating the mean flow through the pass, reveals the strong seasonality of the flow—weakest in summer (6.2 cm s^-1^) and strongest in the cold months (21.3 cm s^-1^). Maximum correlation of flow through Unimak Pass occurs with alongshore (toward 225°) winds [24]. A linear regression on the daily flow through Unimak Pass (U; cm s^-1^) and the alongshore (toward 225°) NARR winds (W; m s^-1^) provided the following relationship:

U=13.0−2.4 W (1)

with an R^2^ = 0.45. Using SST anomalies from a location just upstream (54.625°N, 161.125°W, star in Fig 1) of Unimak Pass, the derived flow (U) into the Bering Sea was color-coded with the temperature of the water (Fig 4C). During March–May each year (2014–2016) northward flow of Gulf of Alaska water through Unimak Pass was largely positive and varied on time scales of days to weeks. However, while warm anomalies were sporadic in March–April 2014, warm conditions were persistent in spring 2015 and 2016 (Fig 4C). In addition, the northward flow of warm water through Unimak Pass was more persistent in March 2015 than in 2014 or 2016, and this was the month with particularly cold north winds in the Bering Sea (Fig 4A).

**Table 1:** Deployment period of moorings deployed in Unimak Pass.

Deployed	End of Record	Length of record (days)
25 March 1980	14 August 1980	142
21 February 1995	18 January 1996	335
26 September 1996	25 September 1997	365
7 May 2001	19 June 2001	44
13 May 2002	1 August 2003	80
8 May 2014	30 May 2014	23

Moorings were deployed at approximately 54.32°N, 164.77°W and measured bottom flow and temperature and salinity.

Strong, positive temperature anomalies developed in the northeast Pacific basin in winter of 2014 [23]. The anomalies were strongest (>2°C) in the basin off the Oregon and Washington coast. During the summer of 2014, the anomaly expanded shoreward, and by autumn, the SST anomalies on the shelf were ~3°C from California northward to the Alaskan coast and the Gulf of Alaska [25]. In 2015, the SST on the shelf to the east of Unimak Pass were particularly warm, and it is this heat that was evident in Fig 4C as this water was advected into the Bering Sea and onto the shelf. While SST in the coastal Gulf of Alaska cooled in 2016, it was still warmer than normal [26].

**Fig 9:**
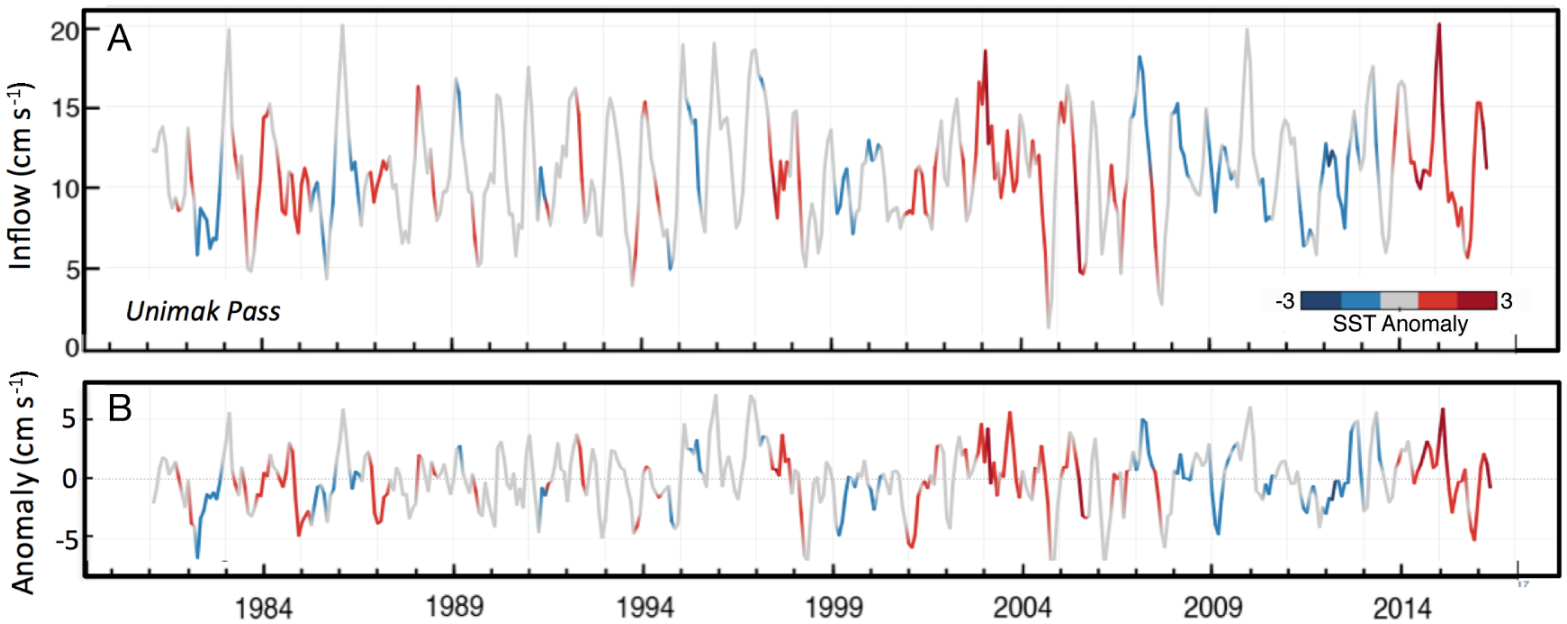
Flow through Unimak Pass. (top panel) Monthly mean derived current through Unimak Pass (Eq 1). The line is color-coded to indicate the SST anomaly upstream near the Shumagin Islands (star in Fig 1, 54.625°N, 161.125°W). Positive indicates inflow into the Bering Sea. (bottom panel) The monthly mean anomaly for flow through Unimak Pass.

An examination of the monthly-averaged flow through Unimak Pass (Fig 9; color-coded with monthly-averaged SST anomalies from the Gulf of Alaska, star in Fig 1) indicates that January–February 2015 had particularly strong flow from the Gulf of Alaska into the Bering Sea. In addition, SST in the Gulf of Alaska was particularly warm. Only in one other year, 2003, out of 37 years, was anomalous warm water advected onto the Bering Sea shelf in winter, and during that year the flow was weaker than what was observed in 2015. That year (2003) was also characterized with a low concentration of sea ice on the southern shelf (Fig 2). In 2016, SST remained warm, but the flow into the Bering Sea was weaker, especially in the winter months.

## Final comments

The presence of sea ice in March and April determines whether the southern Bering Sea will be warm or cold. Our analysis indicates that the more persistent the ice, the colder the depth averaged temperature at M2. In addition, in years with no sea ice during the winter and early spring, the amount of heat in the water during the previous summer strongly influences the temperature in the following year. Finally, the low ice extents in 2014 and 2016 appeared to be primarily a result of anomalously weak northerly winds, but in 2015 strong northward flow of warm Gulf of Alaska water through Unimak Pass likely contributed to limiting ice extent on the southern shelf. This warm water resulted from the positive temperature anomalies that dominated the coastal Gulf of Alaska in 2015. With the available data, it is not possible to determine whether the warm ocean temperatures in the Gulf of Alaska or the magnitude of the northward flow was more important in limiting ice over the southeastern Bering Shelf, but we suggest that it was a combination of both strong transport and warm ocean temperatures in January-March together that limited sea-ice advection.

This second occurrence of consecutive low-ice/warm-ocean years lends support to the paradigm that the Bering Sea has shifted from a system with high year-to-year variability to a system of stanzas–multiple years of warm followed by multiple years of cold. It is not understood what may have caused such a shift, or if it is just a temporary change, but, hopefully, examination of the large-scale climate models can provide insight into this question.

While 2014–2016 were similar to 2001–2003 (the first three years of the previous warm period) in limited ice extent, warm ocean conditions in the vicinity of M2, and localized high nitrite values, there were some subtle differences. At M2, the timing of the spring chlorophyll bloom was earlier (~2 weeks) in 2014 and 2016 than in 2001–2003, while at M4 the timing was more comparable between the two periods [27]. While the maximum ice extent in 2001–2003 reached 58°N at least briefly each year, it retreated earlier than in 2015 and 2016 (Fig 4). Of these six years, 2015 had the most extensive ice that persisted near M4 for approximately two months, resulting in a more extensive cold pool observed by *Duffy-Anderson et al*. [21]. The warm water extended farther north in the earlier warm period (2001–2003), likely narrowing the transition zone between the warm southern shelf and the cold northern shelf which begins at ~60°N [6]. Each of these (timing of sea-ice retreat, location of the transition zone and the timing of the spring phytoplankton bloom) likely has an effect on the ecosystem.

This latest string of three warm years apparently ended this year (2017). In March, sea ice had covered M4, and the ice edge was near or at M2 on 13 March 2017 (http://www.weather.gov/afc/ice), but by June 2017 the depth-averaged temperatures at M2 were average, indicating that ocean temperature near M2 in 2017 (like the 2006 transition between the previous warm and cold stanzas) will likely be average. With this end of a warm stanza, questions arise. Will the Bering Sea continue in a pattern of warm/cold stanzas? On a broader note, if the southern Bering Sea has shifted from high interannual variability to a pattern of stanzas of warm and cold years, what will be the impact on the Bering Sea ecosystem? Specifically, will these three years suffice to examine one of the primary hypotheses that resulted from the Bering Sea Program: that multiple consecutive years of little or no sea ice on the southern shelf would result in poor young-of-the-year pollock survival in the second and succeeding years [7].

## Supporting information

S1 Data NOAAEcoFOCI temperature. Moored temperature data presented from 2014–2016.
(ZIP)

S2 Data NOAAEcoFOCI chlorophyll. Moored chlorophyll fluorescence data presented in Fig 5.
(ZIP)

S3 Data NOAAEcoFOCI 2003 to 16 bottom nitrite. Nitrate concentrations collected on the Bering Sea shelf (2013–2016) and presented in Fig 7.
(XLSX)

S4 Data NOAAEcoFOCI 2016 prawler experimental chlorophyll. Chlorophyll fluorescence data collected by prowler and presented in Fig 6.
(XLSX)
